# Navigating Change, Sustaining Impact: GHSP’s Mission in a Transformed Landscape

**DOI:** 10.9745/GHSP-D-25-00050

**Published:** 2025-12-31

**Authors:** Stephen Hodgins, Ruwaida M. Salem, on behalf of the GHSP Editorial Team

**Affiliations:** a School of Public Health, University of Alberta, Edmonton, Canada. Editor-in-Chief of *Global Health: Science and Practice* Journal, Baltimore, MD, USA.; b Johns Hopkins Center for Communication Programs, Baltimore, MD, USA. Managing Editor of *Global Health: Science and Practice* Journal, Baltimore, MD, USA.; c Members of the GHSP Editorial Team: John Borrazzo, Bruce Cogill, Matthews Mathai, Elaine Menotti, Susan Ontiri, Jim Ricca, Abdulmumin Saad, Madeleine Short Fabic, and Rajani Ved.

## Abstract

GHSP remains resilient in the face of funding challenges following the loss of USAID support. This issue reflects our continuing commitment to delivering valuable open-access knowledge to improve health services and programs.

Here at GHSP, we’re happy to share with you a new issue that showcases innovative solutions to address critical public health challenges around the world, from teleconsultation services that saved lives during COVID-19 in rural Nepal^1^ to a comprehensive quality improvement initiative that significantly reduced maternal mortality in Brazilian hospitals.^2^

We don’t take for granted our ability to bring you this issue. As most readers are aware, earlier this year Johns Hopkins Center for Communication Programs (CCP) lost its funding for GHSP from USAID.^3^ For a journal like ours that operates on the Diamond Open Access model, with no fees for readers or authors, this loss has left us in an especially challenging position.

The abrupt cuts to U.S. government-supported foreign assistance and reductions in funding by other governments that have been major supporters of global health are drastically impacting public health,^4^ particularly in the low- and middle-income countries that have been most heavily reliant on external support. In this time, more than ever, we believe strong partnerships across sectors and countries, rooted in solidarity and based on mutual respect, trust, and shared purpose, is a necessary ingredient to achieving health for all in our interconnected global world. We also think it’s important to continue *sharing lessons about what’s worked well, what hasn’t, and why*—as we implement and adapt our programs and services to meet the needs of the whole population, particularly the most vulnerable, and to improve population health outcomes. This has been, and will continue to be, GHSP’s core mission. We firmly believe that despite the profound changes unfolding across the global health landscape, there remains a critical need for a journal like GHSP.

We deeply value the support of our broad GHSP community—our readers, authors, and reviewers. In response to the new circumstances we face, we continue to streamline our procedures to deliver high-quality content more efficiently, without introducing any fees for readers and authors. We were excited to announce earlier this month that GHSP has joined the Open Journals Collective, a groundbreaking initiative that aims to support sustainable, community-driven open access publishing.^5^ CCP has continued to stand by the journal and we’re grateful for the generous individual donations we have received from many of our supporters. We are also actively engaging with potential donors to help us sustain this work and welcome any promising leads you may wish to share.

After a hiatus of several months earlier in the year, we have reactivated our editorial group and have been working through the backlog of submissions received up until January 2025. This issue reflects that progress, and we are committed to continuing this momentum into 2026 as we process remaining submissions. We also hope to reopen the journal to new submissions early in 2026 and will share further updates as plans are finalized.

While the environment in which we work has changed dramatically, the need to keep learning from program experience and continuously improve remains undiminished. The articles in this issue exemplify why GHSP’s mission remains as vital as ever—the articles showcase the ingenuity, dedication, and evidence-grounded solutions emerging from health systems worldwide, proving that creative problem solving to strengthen programs is possible even in the face of uncertainty and more limited resources. The need for rigorous evidence and accessible communication on public health program experience has never been greater—and with your support and in collaboration with current and new like-minded partners, GHSP will continue to serve as a bridge connecting practitioners, managers, decision-makers, and policymakers to one another and to the knowledge needed to strengthen public health programs globally.

## Notes

**Peer Reviewed**
